# Oxylipins‐Omics Combine With Single‐Molecule Analysis Identifies 13(S)‐HODE Positive Extracellular Vesicles as a Diagnostic Biomarker for AFP‐Negative Hepatocellular Carcinoma

**DOI:** 10.1002/jev2.70368

**Published:** 2026-09-17

**Authors:** Yu Wang, Qiaoting Wu, Huixian Lin, Xiaoqing Jiang, Zhenxun Wang, Chao Yang, Rong Wang, Bo Ma, Yingxue Shen, Jiawei Li, Jiaming Chen, Wanyang Sun, Rongrong He, Lei Zheng, Chunchen Liu, Xin Zhang

**Affiliations:** ^1^ Department of Laboratory Nanfang Hospital Southern Medical University Guangzhou China; ^2^ Guangdong Provincial Key Laboratory of Single‐cell and Extracellular Vesicles Nanfang Hospital Southern Medical University Guangzhou P. R. China; ^3^ Department of Hepatobiliary Surgery Nanfang Hospital Southern Medical University Guangzhou P. R. China; ^4^ State Key Laboratory of Bioactive Molecules and Druggability Assessment College of Pharmacy Jinan University Guangzhou China; ^5^ Intensive Care Unit Nanfang Hospital Southern Medical University Guangzhou China; ^6^ Department of Laboratory Medicine Ganzhou Hospital‐Nanfang Hospital Southern Medical University Ganzhou P. R. China

**Keywords:** 13(S)‐HODE, biomarker, extracellular vesicles, hepatocellular carcinoma, liquid biopsy, machine learning, oxylipins

## Abstract

Efficient diagnostic biomarkers enable early detection of hepatocellular carcinoma (HCC), which improves survival. Circulating extracellular vesicles (EVs) in plasma, as a noninvasive diagnostic carrier, have caused widely concern. Here, a novel oxylipidomic profiling was used to analysis the HCC patients’ tissue‐derived EVs (TD‐EVs) cargo and revealing differentially expressed oxylipins (vs. adjacent tissues, *p*<0.05), which undetected in bulk tissues. The hub oxylipins 13(S)‐HODE discovered in TD‐EVs was validated in plasma derived EVs (PD‐EVs) by using single‐molecule analysis platform, showing superior efficacy in AFP‐negative HCC patients (AUC = 0.8474 vs. 0.7627 in AFP‐positive). Clinically, machine learning integrating EV‐derived 13(S)‐HODE with routine clinical parameters of HCC patients was developed to optimize diagnostic classification. The machine learning model, LightGBM, achieved outstanding performance: AUC_mean = 0.962, Sensitivity = 0.933 (0.660–0.997), Specificity: 0.957(0.760–0.998), 95%CI: 0.958–1.000 in multi‐group classification of HCC diagnostics, demonstrating the enhanced diagnostic efficiency of 13(S)‐HODE positive EV subpopulation with other clinical data. This study firstly establishes EV‐derived 13(S)‐HODE as a concordantly biomarker across tissue and plasma sources. Furthermore, single‐molecule analysis platform offers it a highly sensitive diagnostic performance for HCC, particularly valuable for AFP‐negative HCC subgroup.

## Graphical abstract

Our work identifies EV‐derived 13(S)‐HODE as a novel biomarker for HCC, establishes a single‐molecule platform for EV lipid analysis, and constructs a high‐performance diagnostic model by integrating multi‐dimensional data, offering new potential strategies for early monitoring and precise diagnosis of HCC.2

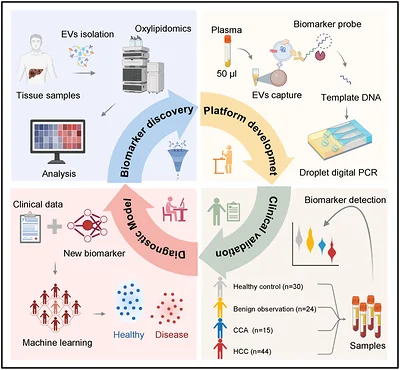

## 1 Introduction

Extracellular vesicles (EVs), 30–150 nm nanovesicles of endosomal origin, are a promising class of liquid biopsy biomarkers (Théry et al. 2018; Welsh et al. 2024). Commonly, EVs are rich in selectively sorted biomolecule cargos including nucleic acids, lipids, and proteins, which play a critical role in mediating intercellular communication under both physiological and pathological conditions, reflecting the current tumour status (Casanova‐Salas et al. 2024; Crescitelli et al. 2025; Hoshino et al. 2020; Liu et al. 2025; Shah et al. 2018; Théry et al. 2018, Welsh et al. 2024; Zhu et al. 2023). Notably, EVs derived from solid tumors are actively released into the peripheral circulation at concentrations exceeding 10^9^ vesicles per milliliter, providing a substantial and accessible source of material for downstream analyses and powerful tools for liquid biopsy (Hoshino et al. 2020). Previous study revealed that proteomic profile of EV particles in human samples from tissue explants significantly exhibited conformity with plasma, confirmed circulating EVs as ideal diagnostic tools to define multiple solid tumour sources (Casanova‐Salas et al. 2024; Crescitelli et al. 2025; Hoshino et al. 2020). Our group also previously demonstrated the prognostic significance and functional relevance of circulating tumor‐derived EV cargo in regulating tumor progression, immune modulation, and the status of primary tumour (Wu et al. 2025; Zhang et al. 2021; Zhang et al. 2025)^.^ These studies on the intersectional molecules between solid tumors and blood EVs holds significant importance and value for the discovery and translation of novel biomarkers. However, the biological concordance between tissue derived EVs and circulating EVs in primary tumor remains inadequately explored.

Hepatocellular carcinoma (HCC) constitutes 75%–85% of primary liver cancers, ranking as the sixth most prevalent malignancy and third leading cause of cancer‐related deaths globally (Siegel et al. 2026). Early diagnosis drastically improves prognosis: patients receiving curative resection at early stages exhibit >50% 5‐year survival, contrasting with < 5% for advanced cases (Lehrich et al. 2024; Wang et al. 2025). The current early screening system for HCC mainly relies on liver cirrhosis as the screening window, utilizing abdominal ultrasound combined with alpha‐fetoprotein (serum AFP > 20 µg/ml as the baseline) and PIVKA‐II for surveillance (Donne and Lujambio 2023; Greten et al. 2023). However, serum AFP levels remain within the normal range in 15%–30% of patients with advanced HCC (Tayob et al. 2023). Studies also have shown that the combination of current diagnostic strategies (AFP + PIVKA‐II) yields a detection rate of approximately 71% for HCC, indicating a significant inadequacy in efficacy (Ricco et al. 2020; Tayob et al. 2023). Thus, developing a better liquid biopsy‐based strategies remains an urgent clinical priority.

As the central organ for lipid metabolism, liver undergoes profound lipidomic reprogramming during carcinogenesis, wherein tumor cells dysregulate lipid synthesis, degradation, and oxidation to fuel proliferation (Liu et al. 2025). Oxylipins, as an oxygenated metabolite of polyunsaturated fatty acids (PUFAs)—have been implicated in various pathological processes in cancer, through stimulating tumor growth and progression, promoting cell proliferation, preventing apoptosis, and increasing invasion and metastasis (Guri et al. 2017; Hateley et al. 2024). Therefore, monitoring the types and levels of oxylipins may serve as an effective strategy for predicting tumor status. Furthermore, oxylipins are widely present in biological fluids, existing either in free form or bound to proteins such as albumin (Ning et al. 2022). They can also be embedded within EVs or anchored to membrane surfaces via phospholipid esters, indicating that EVs serve as an important carrier (Biagini et al. 2024; Pizzinat et al. 2020). Our group also previously demonstrated that EVs can carry and deliver membrane‐derived contents and effectively protecting them from degradation (Wu et al. 2025; Zhang et al. 2021; Zhang et al. 2025). Although HPLC‐MS/MS‐based studies demonstrate the biomarker potential of oxylipins across various cancers, including breast, colorectal, lung, and HCC (Chistyakov et al. 2023; Surmiak et al. 2020), research focusing on oxylipins within EVs remains relatively limited. This gap may be largely attributed to the technical challenge of precisely detecting EVs against the complex background of circulating lipid biomolecules. To enable more sensitive and specific analysis of EV biomarkers, we previously developed a series of droplet digital platforms for single‐EV detection (Liu et al. 2018; Liu et al. 2023; Liu et al. 2021). These platforms effectively revealed EV protein and nucleic acid profiles and demonstrated substantial translational potential in cancer biomarker research. Given their flexible modifiability for detecting various markers, we further developed an advanced droplet microfluidics‐based single‐molecule analysis technology for sensitive plasma‐based lipid biomarker discovery, thereby facilitating the screening and validation of EV biomarkers, including promising oxylipins.

In this study, we proposed a mass spectrometry‐based analysis of EVs from HCC solid tumour, identifying the EV subpopulation encapsuled oxylipin 13(S)‐HODE, known as metabolite of 15‐LOX‐1 (Cabral et al. 2014; Shin and Byun 2024; Yuan et al. 2010; Zuo and Shureiqi 2011). Herein, we firstly established a novel droplet microfluidics platform for ultrasensitive EV membrane oxylipin analysis at single‐molecule resolution to validate the 13(S)‐HODE in plasma EVs. We validated consistent downregulation of 13(S)‐HODE in HCC plasma EVs, demonstrating favorable diagnostic performance for HCC patients (AUC = 0.7992), especially in AFP‐negative patients (AUC = 0.8474 vs. 0.7627 in AFP‐positive). Finally, a machine learning model combines EV‐derived 13(S)‐HODE levels with routine clinical parameters (AUC_mean = 0.962) with LightGBM model, which bridges novel EV biomarkers with conventional clinical data and advances personalized medicine, facilitating biomarker discovery and clinical translation from bench to bedside.

## 2 Materials and Methods

### 2.1 Samples Collection

A total of 7 paired tumor tissues and adjacent normal tissues were collected from patients undergoing HCC resection at Nanfang Hospital between 2022 and 2024. Blood samples were obtained at diagnosis from patients with HCC (*n* = 44), benign hepatobiliary diseases (*n* = 24), and cholangiocarcinoma (*n* = 15). Blood samples were collected from healthy donors used as control (*n* = 30). Informed consent form from participants and the workflow was approved by the Ethics Committee of Nanfang Hospital (Approval No. NFEC‐2022‐056). The inclusion/exclusion criteria and the clinical characteristics are summarized in Table S1‐S2.

### 2.2 EVs Isolation

As previous reported (Wu et al. 2025; Zhang et al. 2021; Zhang et al. 2025), after weighing the tissue (∼300 mg), it was gently sliced into small fragments (2 × 2 × 2 mm) and incubated in DMEM supplemented with 2 mg/ml collagenase D (Yingxin laboratory, YXM07650, China) and 40U/ml DNase I (Solarbio, D8071, China) for 40 min at 37°C. After a filtration step with 70 µm sterile cell filter (Biologix, 15–1070, China), cells and tissue debris were further eliminated by differential centrifugation (300 g, 4°C, 10 min, 2000 g, 4°C, 20 min, 12000 g, 4°C, 30 min) (Eppendorf, Centrifuge 5810 R, Germany) and 0.45 µm syringe—driven filter unit (Merck millipore, SLHV033RS, USA). After these clearing steps, EVs were concentrated by ultracentrifugation (135000 g, 4°C, 150 min) (Beckman Optima L‐80XP ultracentrifuge and Beckman Centrifuge Tubes 355642, Beckman, USA). Then we resuspend EVs with PBS and pure EVs by ultracentrifugation with 10%, 30%, 45% iodixanol density cushion (135000 g, 4°C, 150 min) (Beckman Optima L‐80XP ultracentrifuge and Beckman Centrifuge Tubes 361623, Beckman, USA). Finally, this band was carefully collected and stored at −80°C until subsequent characterization and analysis.

### 2.3 Nanoparticle Tracking Analysis (NTA)

To quantify of the number and size distribution of EVs, the rate of Brownian motion of the particles was determined by ZetaView nanoparticle tracking analyzer (Particle Metrix, ZetaView 8.04.02, Germany) and NanoSight NS300 (Malvern Panalytical, UK). The instrument was cleaned by pure water until the number of detected particles less than 5. The instrument pre—acquisition parameters were set to a sensitivity of 70 and shutter of 70. Samples were diluted in PBS to achieve a particle count in the range of 150 to 200 particles per visual field. EVs samples were usually diluted in PBS with dilution factor 1:1000 and were loaded to the instrument and analyzed.

### 2.4 Antibodies

The antibody was CD63(1:5000, Abcam, ab134045), CD9 (1:1000, Abcam, ab263019), TSG101(1:5000, Abcam, ab125011), GM130 (1:5000, Abcam, ab52649), Goat Anti‐Rabbit IgG H&L (1:10000, bioss, bs‐0295G‐HRP), 13(S)‐HODE polyclonal antibody(1:20, BIOHUB, 78DA10023), Rabbit Anti‐Goat IgG/Gold 10 nm (1:20, Solarbio, K1038R‐G10), Goat IgG polyclonal—Isotype Control (1:20, Abcam, ab37373), FITC Anti‐CD63 antibody (1:5, Abcam, ab18235).

### 2.5 Protein Quantification, Electrophoresis, and Immunoblotting

Protein concentration of isolated EV was measured using the BCA Protein Assay Kit according to the manufacturer's instructions (Beyotime, P0012, China). In order to evaluate the expression of characteristic EVs markers (TSG101, CD63, CD9) and EVs negative control protein GM130, 20 µg of EVs fraction were denaturalized by adding non‐reducing protein loading buffer (NCM, WB2001, China) and by heating the samples at 100°C for 5 min. Then, proteins were separated in a 10% SDS‐PAGE (Beyotime, P0469S, China) and electro‐transferred onto a 0.45 µm polyvinylidene fluoride membrane (Merck millipore, IPVH00010, USA). After blocking with 5% BSA for 1 h at room temperature, membranes were incubated overnight at 4°C with the appropriate primary antibodies. Membranes were then washed 3 times with TBS‐T and incubated with horseradish peroxidase‐conjugated secondary antibodies at a dilution of 1:5000 (in blocking solution) for 1 h at room temperature. After washing the membranes with 1 × TBST for 10 min 3 times, the membranes were incubated with secondary antibody for 1 h at room temperature. The membrane was washed with 1 × TBST for 10 min 3 times and incubated with FDbio‐Dura ECL Kit (Fdbio science, FD8020, China). Finally, the emitted chemiluminescence was visualized and captured with the chemiluminescence instrument (Eblot, N30447, China).

### 2.6 Transmission Electron Microscopy (TEM)

A 10 µL sample aliquot was applied to a copper mesh (EMCN, BZ11023b, China) and allowed to precipitate for 1 min. Excess liquid was subsequently removed using filter paper. Following this, a 10 µL aliquot of uranyl acetate solution (EMCN, GZ02625‐5, China) was applied to the mesh and similarly allowed to precipitate for 1 min, after which excess liquid was again removed with filter paper. The prepared mesh was then air‐dried at room temperature for 5 min prior to examination by transmission electron microscopy (Hitachi, HT‐7700, Japan) at 80 kV.

### 2.7 Oxylipidomics

Proteins were precipitated by adding methanol to tissue and tissue‐derived EV samples at a 1:4 (v/v) ratio. Tissue samples were homogenized prior to precipitation. Targeted oxylipin analysis was performed using an LC‐20AD liquid chromatograph coupled with a Triple Quad 6500+ mass spectrometer. A list of analyzed oxylipins is provided in the Supplementary File. Separation was achieved on an ACQUITY UPLC HSS T3 column (1.8 µm, 2.1 mm × 100 mm) at 0.5 mL/min over 13 min with gradient elution (mobile phases A: acetonitrile/water/formic acid, 45/55/0.01, v/v/v; B: acetonitrile/isopropanol/formic acid, 50/50/0.01, v/v/v). The column temperature was 40°C, and the injection volume was 2 µL. Oxylipins were analyzed in scheduled multiple reaction monitoring mode (60 s detection window) in both positive and negative ion modes, with MS parameters: electrospray voltage, ±4.5 kV; source temperature, 400°C; GS1 and GS2, 55 psi; curtain gas, 35 psi; CAD, Medium. Quantification was performed using ratio metric comparison with internal standards and standard curves per oxylipin class. Data acquisition and processing were conducted with Analyst 1.6.2 and SCIEX OS 3.3.1.43, respectively. Protein concentrations in tissues and EVs were determined using a BCA assay (Beyotime, China) for data normalization.

### 2.8 Single Molecule Analysis Design and Detection

#### 2.8.1 Fabrication of Functionalized Magnetic Beads

To prepare magnetic beads for extracellular vesicle (EV) capture, antibodies against the EV markers CD9, CD63, and CD81 were firstly biotinylated. Briefly, Sulfo‐NHS‐LC‐Biotin (1 mg, A39257, Thermo Fisher Scientific) was dissolved in PBS to a 10 mM stock solution and then diluted 100‐fold with PBS to a working concentration of 0.1 mM. A volume of 7 µL of the biotin working solution was added to 1.65 µL of each antibody. The mixture was incubated at room temperature for 30 min, followed by overnight incubation at 4°C (16 hours). For conjugation, 100 µL of streptavidin‐coated magnetic beads (Dynabeads MyOne Streptavidin T1, 65601, Thermo Fisher Scientific) were aspirated and washed twice with 100 µL of PBS. The biotinylated antibody mixture was diluted to 100 µL with PBS and then incubated with the prepared beads at room temperature for 60 min. The beads were subsequently washed three times with 100 µL of PBS, blocked with 100 µL of 1% bovine serum albumin (BSA) for 30 min, and washed again three times with PBS before being resuspended in 100 µL of PBS. Functionalized polystyrene microspheres (Bangs Laboratories) were prepared identically for scanning electron microscopy (SEM) analysis. All functionalized beads and microspheres were stored at 4°C for further use.

#### 2.8.2 Preparation of Antibody‐Oligonucleotide Conjugates Detection Probes

The conjugation of the linker to oligonucleotides was first validated. A reaction mixture containing 2 µL of FAM‐labeled oligonucleotide (100 µM), 10 µL of 2 × ssDNA buffer (100 mM HEPES, 300 mM NaCl, 2 mM MgCl_2_, 2 mM MnCl_2_, pH 8.0), and 4.5 µL of ultrapure water was prepared. Then, 3.5 µL of Linker (1 mg/mL, 984538, J&K Scientific) was added. The mixture was incubated at 37°C for 1 h. Subsequently, to form the antibody‐oligonucleotide conjugates, 5 µL of the Linker‐oligonucleotide product was mixed with 3 µL of the 13(S)‐HODE antibody (BIOHUB, 78DA10023) and incubated at 4°C for 1 h. The resulting conjugates (13(S)‐HODE‐Ab‐oligo) were diluted to a final volume of 30 µL with PBS and stored at 4°C until use.

### 2.9 Droplet Digital PCR (ddPCR) Construction

All oligonucleotides (primers and probes) were synthesized by Sangon Biotech (Shanghai, China) and dissolved in 1× TE buffer. Sequences and final concentrations are detailed in Table S3. A 20 µL PCR reaction mixture was prepared, containing: 10 µL of 2× ddPCR Universal Probe Mix (Probe dPCR Universal Kit, Thunderbio), 1.8 µL of forward primer, 1.8 µL of reverse primer, 0.6 µL of 10× probe, 3.8 µL of nuclease‐free water, and 2 µL of template‐containing reaction liquid. Droplets were generated by loading the reaction mixture along with droplet generation oil (Thunderbio) into the droplet generation chip. The generated droplets were transferred to an eight‐tube strip for amplification on a thermal cycler (Bioer). The optimized thermal cycling conditions were initial denaturation at 94°C for 5 min; 45 cycles of denaturation at 94°C for 30 s, and combined annealing/extension at 58°C for 2 min; followed by a final hold at 4°C. The amplified droplets were analyzed using the ExoStar Droplet Reader (Thunderbio) with a dedicated analysis chip (Probe dPCR Universal Chip Kit, Thunderbio). Target concentration was calculated automatically by the instrument software based on Poisson statistics.

### 2.10 Scanning Electron Microscopy (SEM)

For SEM analysis, 2 µL of CD9/CD63/CD81‐functionalized microspheres were incubated with 50 µL of pre‐treated human plasma in a total volume of 150 µL PBS at room temperature for 1 h to capture EVs. The samples were washed twice with 50 µL PBS and resuspended in 50 µL PBS. The captured EVs were fixed with a mixture of 2% paraformaldehyde and 2.5% glutaraldehyde for 2 h. Dehydration was performed using a graded ethanol series (50%, 70%, 80%, 90%, and 100%), with rinsing in PBS after each step to prevent salt crystallization. Ethanol was then replaced with isoamyl acetate. Samples were freeze‐dried overnight, and a thin gold layer was sputter‐coated onto their surface using an Apero 2S HiVac system (Thermo Fisher Scientific) to enhance conductivity for SEM imaging.

### 2.11 Flow Cytometry Analysis

To validate the functionality of the CD9/CD81‐functionalized magnetic beads, 2 µL of beads were mixed with 10 µL of a FITC‐labeled anti‐CD63 primary antibody. This mixture was then split into two groups: a negative control, brought to 50 µL with PBS, and a positive control, brought to 50 µL with a dilution of plasma derived EVs. After incubation at room temperature for 1 h in the dark, the samples were washed three times with 50 µL of PBS and analyzed on a flow cytometer (Cytoflex, N31376).

### 2.12 Immunogold Labeling

To verify the localization of 13(S)‐HODE on EV membranes, EVs were mixed with 4% paraformaldehyde at a 1:1 ratio. A copper grid (BZ11023b, ZJKY) was placed face‐down onto the mixed droplet and incubated for 20 min. The grid was then sequentially washed with 100 µL droplets of PBS, quenched with 50 mM glycine, and blocked with 5% BSA. Primary incubation was performed using an anti‐13(S)‐HODE antibody (78DA10023, BIOHUB) for 40 min at room temperature, followed by washing with 0.1% BSA. The grid was then incubated with a 10 nm colloidal gold‐conjugated rabbit anti‐goat IgG secondary antibody (K1038R‐G10, Solarbio) for 40 min at room temperature. After sequential washing with 0.5% BSA and PBS, the grid was fixed with 1% glutaraldehyde for 2 min and rinsed with distilled water. Negative staining was performed with 2% uranyl acetate (GZ02625‐5, Henan Xinrui) for 90 s. Excess stain was removed with filter paper, and the grid was air‐dried. Imaging was conducted using a transmission electron microscope (HT‐7700, Hitachi, Japan) at 80 kV. For the negative control, the primary antibody was replaced with a goat IgG isotype control (ab37373, Abcam) at the same dilution.

### 2.13 EV Capture and 13(S)‐HODE Detection

For the detection of 13(S)‐HODE on plasma EVs, 2 µL of CD9/CD63/CD81‐functionalized magnetic beads were added to 50 µL of plasma and incubated at room temperature for 1 h to capture and enrich EVs. Then, 50 µL of PBS was added, and the sample was washed twice to remove unbound plasma components. Meanwhile, the 13(S)‐HODE‐Ab‐oligo conjugates were diluted to a final concentration of 60 pM. Then, 2.5 µL of this conjugate solution was added to the purified EV‐bead complex. The volume was adjusted to 50 µL with blocking buffer, and the mixture was incubated at room temperature for 1 h. After incubation, the sample was washed four times with 50 µL of washing buffer. To release the oligonucleotide barcodes, 0.1 µL of RNase A/T1 was added, and the volume was adjusted to 50 µL with PBS, followed by incubation at room temperature for 20 min. Finally, the supernatant was collected and analyzed by ddPCR for absolute quantification of the released oligonucleotides, which directly correlates to the abundance of the 13(S)‐HODE target on the captured EVs.

### 2.14 Enzyme‐linked Immunosorbent Assay

The concentration of 13(S)‐HODE in plasma was quantified using a competitive enzyme‐linked immunosorbent assay (ELISA). Measurements were performed according to the manufacturer's protocol for the human 13(S)‐HODE ELISA kit (COIBO, CB12258‐Hu, China). Absorbance was measured at 450 nm using a microplate reader (Tecan Spark, N34227, China).

### 2.15 Model Training Pipeline

All analyses were implemented in Python with pandas, NumPy, scikit‐learn, XGBoost and LightGBM. Three‐fold cross‐validation was performed: the dataset was split into three disjoint subgroups; two subgroups constituted the training set per fold, and the remaining one was held out as an independent test set. Nine classifiers (LR, LDA, MLP, linear/rbf SVM, RF, AdaBoost, GBC, XGBoost, LightGBM) were evaluated. Model hyperparameters were preliminarily tuned on a separate preliminary dataset and then fixed throughout the entire three‐fold cross‐validation process. No parameter adjustment was conducted within individual validation folds to avoid data leakage. Each model was trained exclusively on fold‐specific training data before generating predictions on unseen test samples. Classification labels, predicted probabilities, and feature importance metrics (for tree‐based algorithms) were exported as CSV files.

### 2.16 Data Analysis

The ExoStar software (Thunderbio) was used to analyze data obtained from ddPCR assays. GraphPad Prism 10.1.2 was employed to assess the statistical significance of the data. Mann‐Whitney test was used for unpaired samples. Wilcoxon matched pairs signed rank test were used for paired samples. Continuous variables are expressed as mean ± standard deviation (SD). *p* < 0.05 is considered statistically significant. Multivariate statistical analysis (PCA and OPLS‐DA) and Pathway Analysis were performed on the lipidomic data using the MetaboAnalyst 6.0 online platform. To explore potential connections between key oxylipins and HCC‐related genes, an integrative gene‐lipid interaction network was constructed using the “Network Analysis” module of MetaboAnalyst 6.0. HCC differentially expressed genes were derived from GEO datasets GSE144269 and GSE14520 (screened with |log2FC| > 1, adj. *p* < 0.05), and high‐confidence HCC target genes were obtained from the GeneCards database (relevance score > 15).

## 3 Results

### 3.1 EVs Isolation and Characterization

**FIGURE 1:**
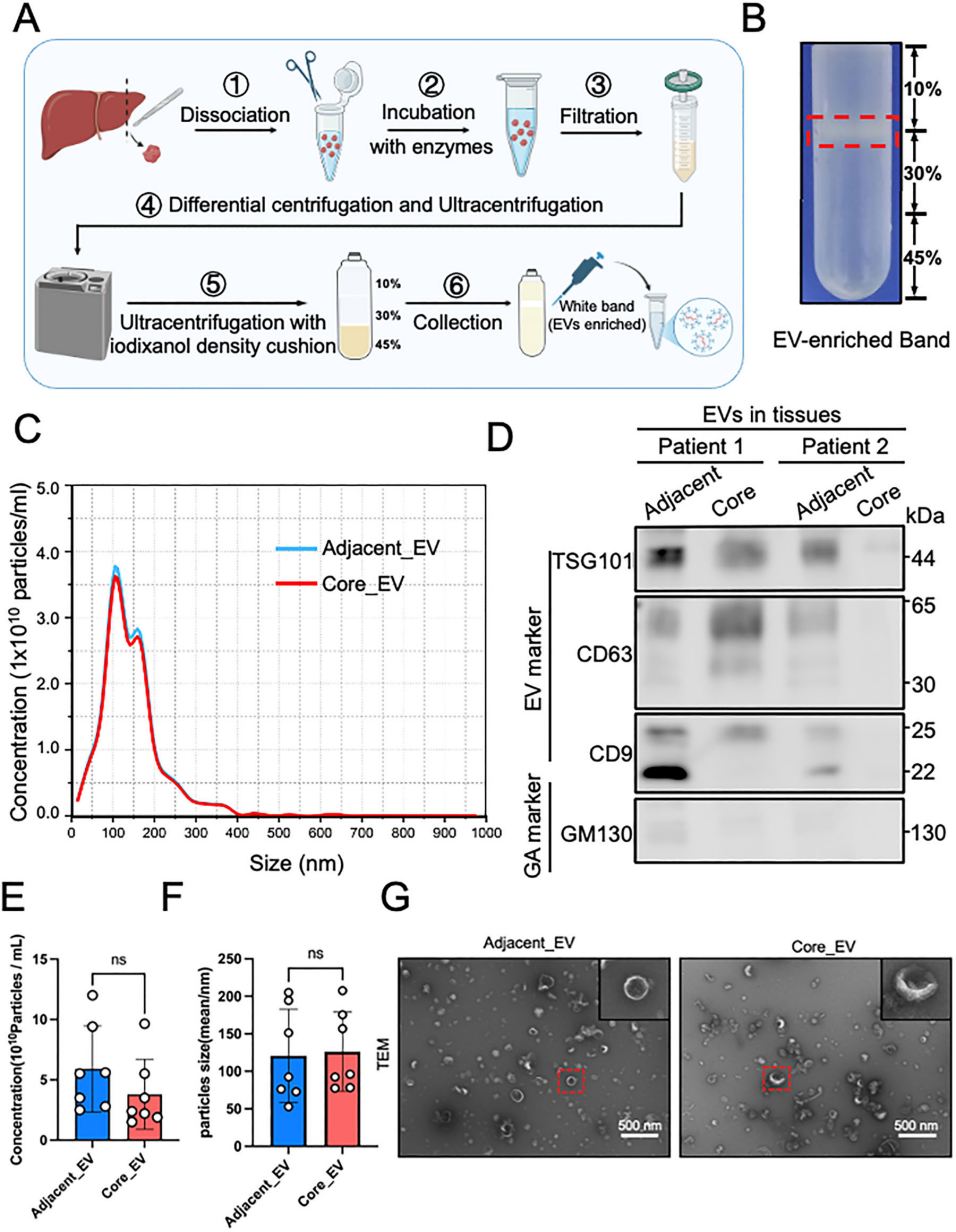
EVs isolation and characterization. (A) Schematic diagram of the EV isolation workflow. (B) Representative image of the EV‐enriched band after ultracentrifugation. (C) Representative image of nanoparticle tracking analysis (NTA) of isolated EVs. (D) Western blot analysis of EV and Golgi apparatus (GA) markers. (E‐F) Concentration (particles/mL) and mean size of EVs particles across measured samples. ns, non‐significant. (G) Transmission electron microscopy (TEM) image of isolated EVs. Scale bar, 500 nm.

Firstly, EVs from adjacent non‐tumorous tissues and core tumor tissues obtained from HCC patients were isolated. As detailed workflow showed in Figure 1, EVs were isolated by using iodixanol density cushion (IDC) combine with ultracentrifugation (Figure 1A), recommend by ISEV2018 and ISEV2023 (Théry et al. 2018; Welsh et al. 2024). Therefore, EV‐enriched band was visible in the supernatant following the above centrifugation (Figure 1B). Western blot confirmed positive expression of canonical EV marker proteins (CD9, CD63, and TSG101) and negative expression of Golgi apparatus (GA) marker protein GM130 (Figure 1D). Nanoparticle tracking analysis (NTA) demonstrated a predominant particle size distribution peaking near 105 nm, consistent with the expected size range for EVs, with a concentration of 10^10^ particles/mL (Figure 1E–F). Furthermore, transmission electron microscopy (TEM) analysis revealed that the isolated EVs displayed characteristic cup‐shaped morphology with a double‐layered membrane structure (Figure 1G). Collectively, these results demonstrated the purely isolation of tissue derived EVs (TD‐EVs) from HCC patients.

### 3.2 Oxylipins Profiling in Adjacent‐EVs and Core‐EVs

**FIGURE 2:**
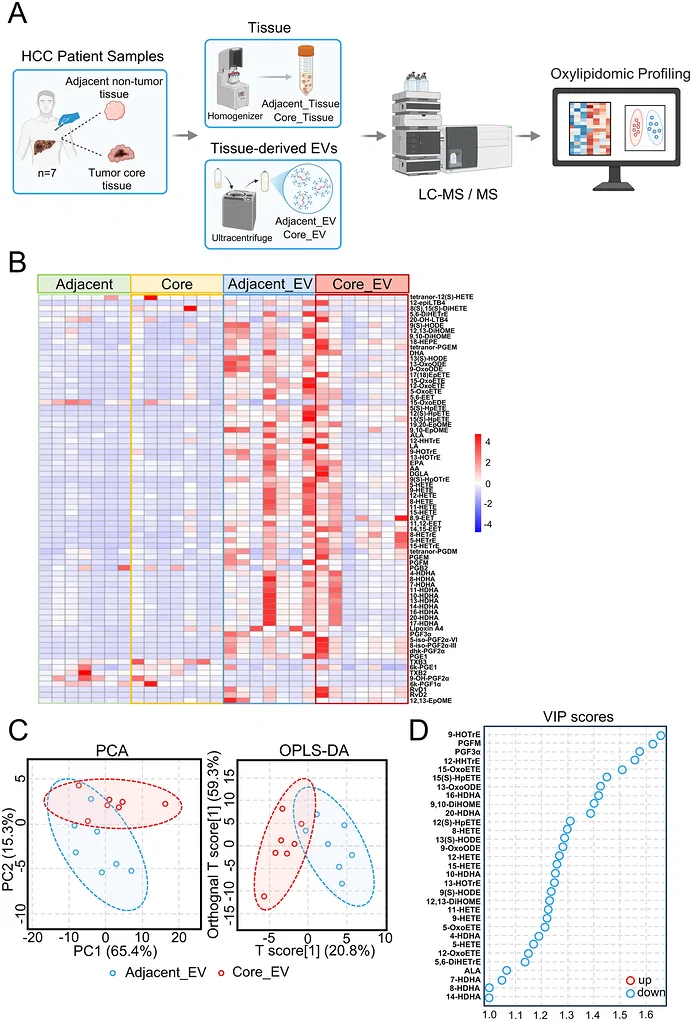
Oxylipins profiling in adjacent‐EVs and core‐EVs. (A) Schematic diagram of mass spectrometry‐based oxylipidomic analysis. (B) Comparison of oxylipin levels between tissue homogenization and EV fractions. (C) Multivariate analysis (PCA and OPLS‐DA) of oxylipin profiles in EVs. (D) Identification of oxylipins contributing to group discrimination.

To investigate the differential expression of EV cargo between core‐ and adjacent‐derived EVs, we using a novelty platform, mass spectrometry‐based oxylipin‐omics, to analysis EV oxylipin from 7 pairs of solid tissues (adjacent and core tissues) and isolated tissue‐derived EVs (TD‐EVs), as detailed in Figure 2A. Notably, the oxylipins exhibited significant enrichment within EVs compared to tissue lysates, which identified 74 distinct oxylipins. The heatmap show that, oxylipin contents were not distinctly different in the solid tissues (the adjacent and the core), while the profile exhibited significantly higher in adjacent‐EVs compared to core‐EVs (Figure 2B), respectively. Both principal component analysis (PCA) and orthogonal partial least squares‐discriminant analysis (OPLS‐DA) between the two EV groups revealed clearly distinct oxylipin signatures, indicating significant differences in metabiotic profiles (Figure 2C). The variable importance in projection (VIP) scores showed the top 31 oxylipins were responsible for this distinction (Figure 2D). Notably, these findings revealing the encapsule oxylipins cargo into EVs may highly selective between the adjacent and the core in HCC human tissues.

#### 3.2.1 13(S)‐HODE Positive EVs act as a Candidate Biomarker in HCC

**FIGURE 3:**
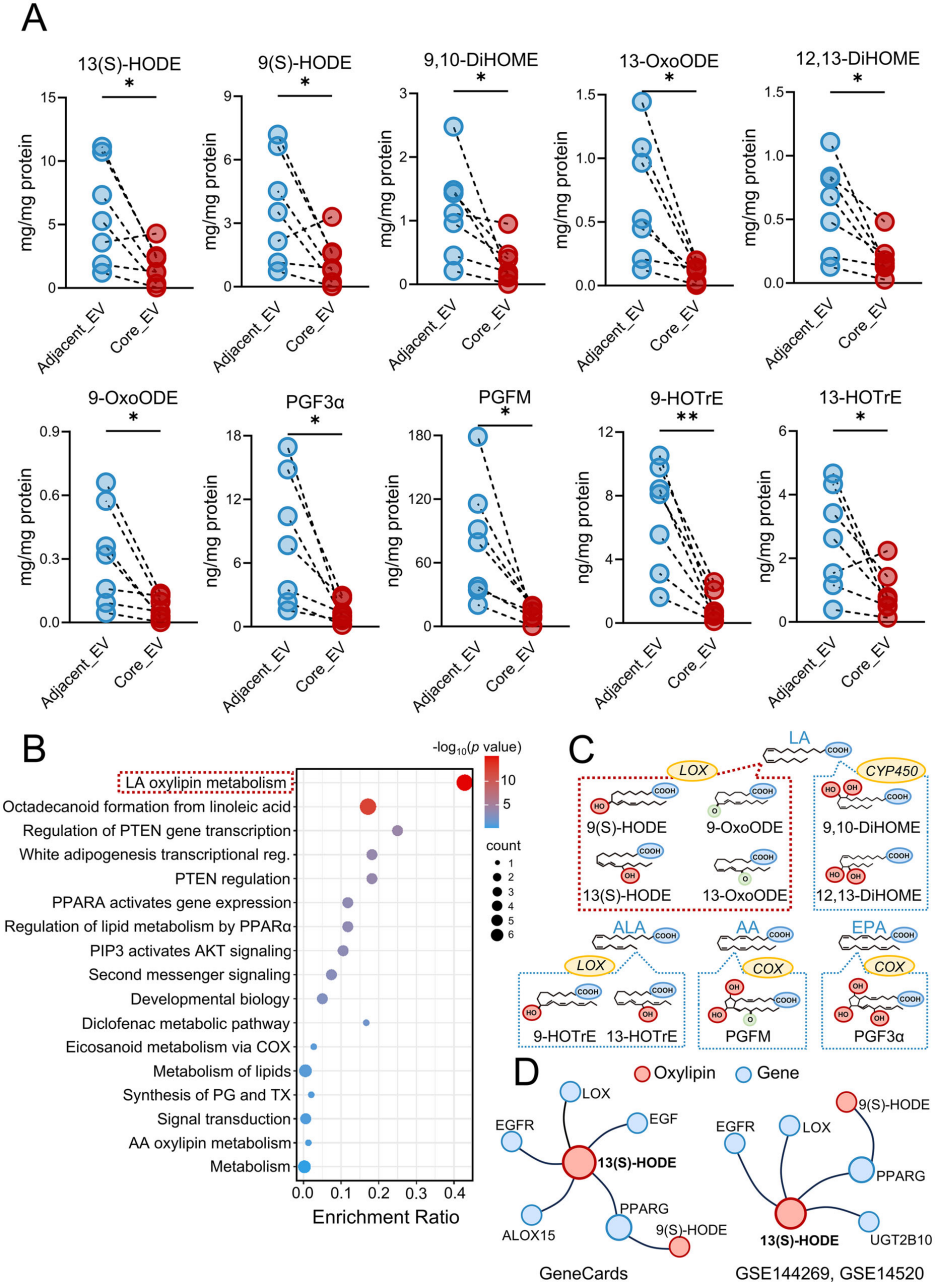
13(S)‐HODE positive EVs act as a candidate biomarker in HCC. (A)Statistical analysis of oxylipin levels between tumor and adjacent tissue derived EVs. *n* = 7. *p < 0.05, **p < 0.01. *p*‐value was calculated using the Wilcoxon statistical test. (B) Pathway enrichment analysis of differential oxylipins. (C) Metabolic origin analysis of the differential oxylipins. (D) Integrated gene‐oxylipin interaction network analysis for core regulatory oxylipin identification.

To determine whether those oxylipins cargos serve as potential tumour biomarker, we further quantified the level of distinct EV oxylipins subtype in total EVs. Among the oxylipins assessed, 10 oxylipins cargo including 13(S)‐HODE, 9(S)‐HODE, 9,10‐DiHOME, 13‐OxoODE, 12,13‐DiHOME, 9‐OxoODE, PGF3α, PGFM, 9‐HOTrE, 13‐HOTrE were significantly reduced in the EV lysates from core‐EVs compared with adjacent‐EVs, while there was not significantly difference in the solid tissues (* *p* < 0.05, ** *p* < 0.01) (Figure 3A). Notably, all these differential oxylipins were among the key lipids previously identified by OPLS‐DA modeling with VIP scores >1 (Figure 2D). In addition, the landscape exhibited the enriched metabolic pathway including the linoleic acid oxylipin metabolism and the PIP3‐mediated AKT signaling pathway, etc. (Figure 3B). Metabolic origin analysis further confirmed that 6 differential oxylipins were direct derivatives of linoleic acid (LA) metabolism (Figure 3C). Furthermore, we constructed a gene‐oxylipin interaction network by integrating three key elements: HCC‐related differentially expressed genes from GEO datasets (GSE144269, GSE14520), key target genes from GeneCards, and the ten critical oxylipins identified in this study. This analysis identified 13(S)‐HODE as a hub oxylipin in the regulated network (Figure 3D). Taken together, these findings suggested that reduced 13(S)‐HODE in EVs may serve as a potential indicator of HCC.

### 3.3 Single‐Molecule Detection of Plasma EV 13(S)‐HODE

**FIGURE 4:**
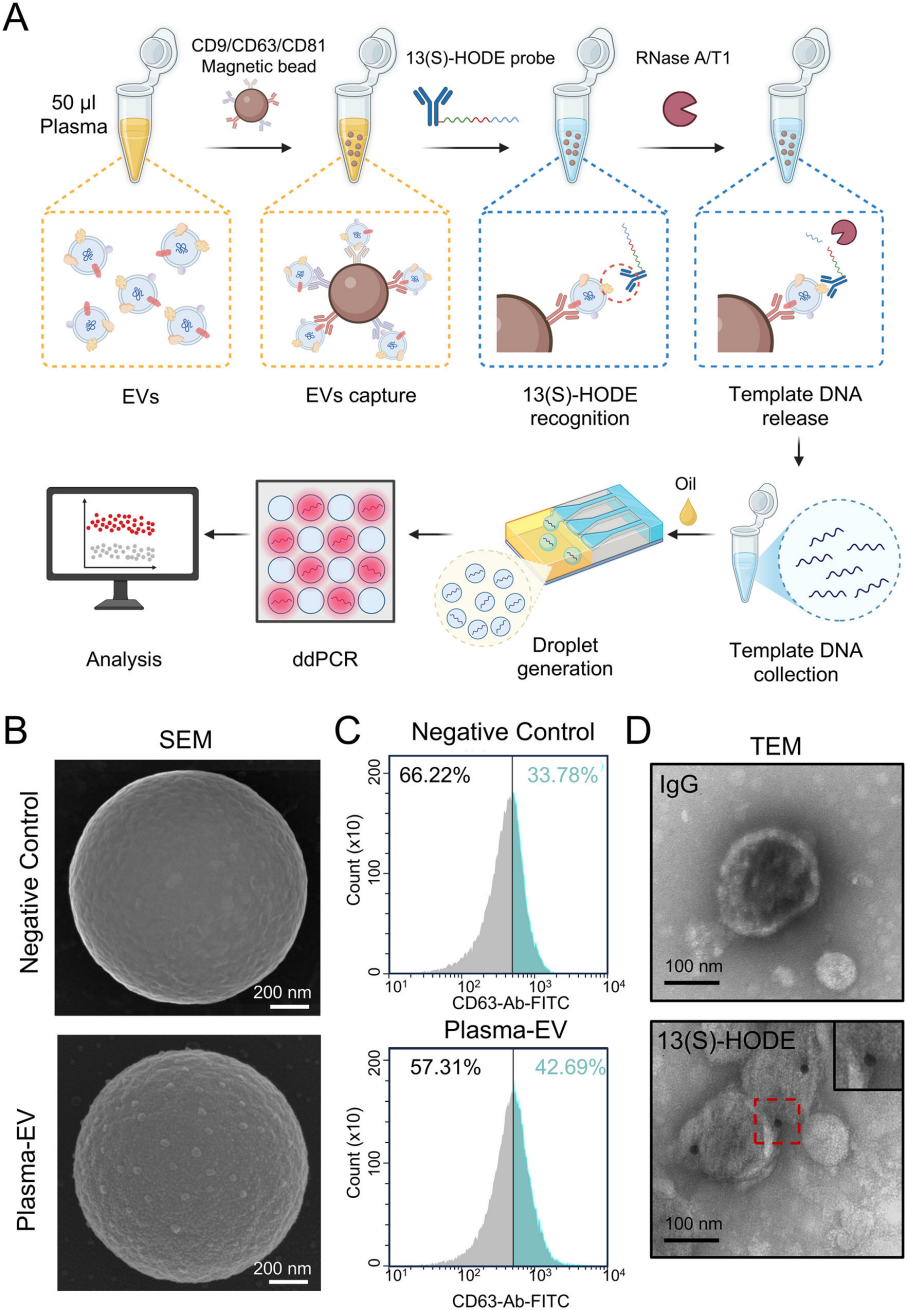
Single‐molecule detection of plasma EV 13(S)‐HODE. (A)Schematic workflow of the custom droplet digital PCR (ddPCR) assay for quantifying EV‐surface 13(S)‐HODE. (B) Scanning electron microscopy (SEM) images of functionalized magnetic beads incubation with plasma EVs versus control. (C) Flow cytometry analysis comparing fluorescence signals of functionalized beads incubated with CD63‐FITC‐labeled plasma EVs versus control. (D) Immunogold labeling of 13(S)‐HODE on EV membrane surface, visualized using Uranyl Acetate (UA) staining and TEM, scale bar: 100 nm.

Mounting evidence suggests that EVs could be used for early cancer detection, prognosis, and to guide therapy (Hoshino et al. 2020). EVs are actively released into the peripheral circulation at concentrations of > 10^9^ vesicles/mL, providing ample material for downstream analyses (Zhang et al. 2021). Herein, to detect minute amounts of distinct EV subpopulation in peripheral, we therefore generate an ultrasensitive EV membrane oxylipins detection platform based on droplet microfluidics at single‐molecule resolution. Specifically, we have innovatively designed a lipid biomarker probe consisting of an antibody conjugated with a breakable DNA strand, enabling both lipid recognition and signal transformation. The released DNA strand would trigger the droplet digital PCR (ddPCR) system to achieve single‐molecule lipid detection. By integrating the antibody‐based specific recognition with the ddPCR ultra‐sensitivity, this platform is expected to facilitate the screening and validation of EVs lipid markers. As detailed in Figure 4A, we would firstly construct the functional beads modified with anti‐CD9/63/81 for EVs capture during the incubation with plasma samples. The captured EVs on beads surface were then specifically recognized by the 13(S)‐HODE antibody‐nucleic acid probe, followed by RNase A/T1‐mediated release of the DNA template end for subsequent ddPCR detection, enabling single‐molecule digital absolute quantification of EV membrane‐located 13(S)‐HODE. Furthermore, to validate the performance of the functionalized beads in capturing plasma derived EVs, CD9/CD63/CD81‐functionalized microspheres were incubated with the liquid and analyzed by SEM. Results revealed EV‐like structures on the surface of the microspheres in the experimental group compared to the negative control (Figure 4B), confirming successful EV capture directly from plasma by the functionalized magnetic beads. Flow cytometry further validated the functionality of the beads: incubation of functionalized beads with plasma EVs labeled with FITC‐conjugated CD63 antibody resulted in an obvious fluorescence signal in the experimental than in the negative control, verifying the bioactivity of the functionalized magnetic beads (Figures 4C and S1 **)**. In addition, immunogold labelling revealed the presence of 13(S)‐HODE on the surface of EVs derived from HCC cells (Figure 4D **),** ensuring its potential binding capability with the 13(S)‐HODE antibody‐nucleic acid probe. Above all, these results confirmed the efficiency of the customized detection platform system for analyzing plasma 13(S)‐HODE positive EVs and continuously contributed to evaluate its performance as a non‐invasive diagnostic biomarker for early HCC in a clinical cohort.

### 3.4 The Diagnostic Performance of Plasma Derived 13(S)‐HODE‐Positive EVs in HCC

**FIGURE 5:**
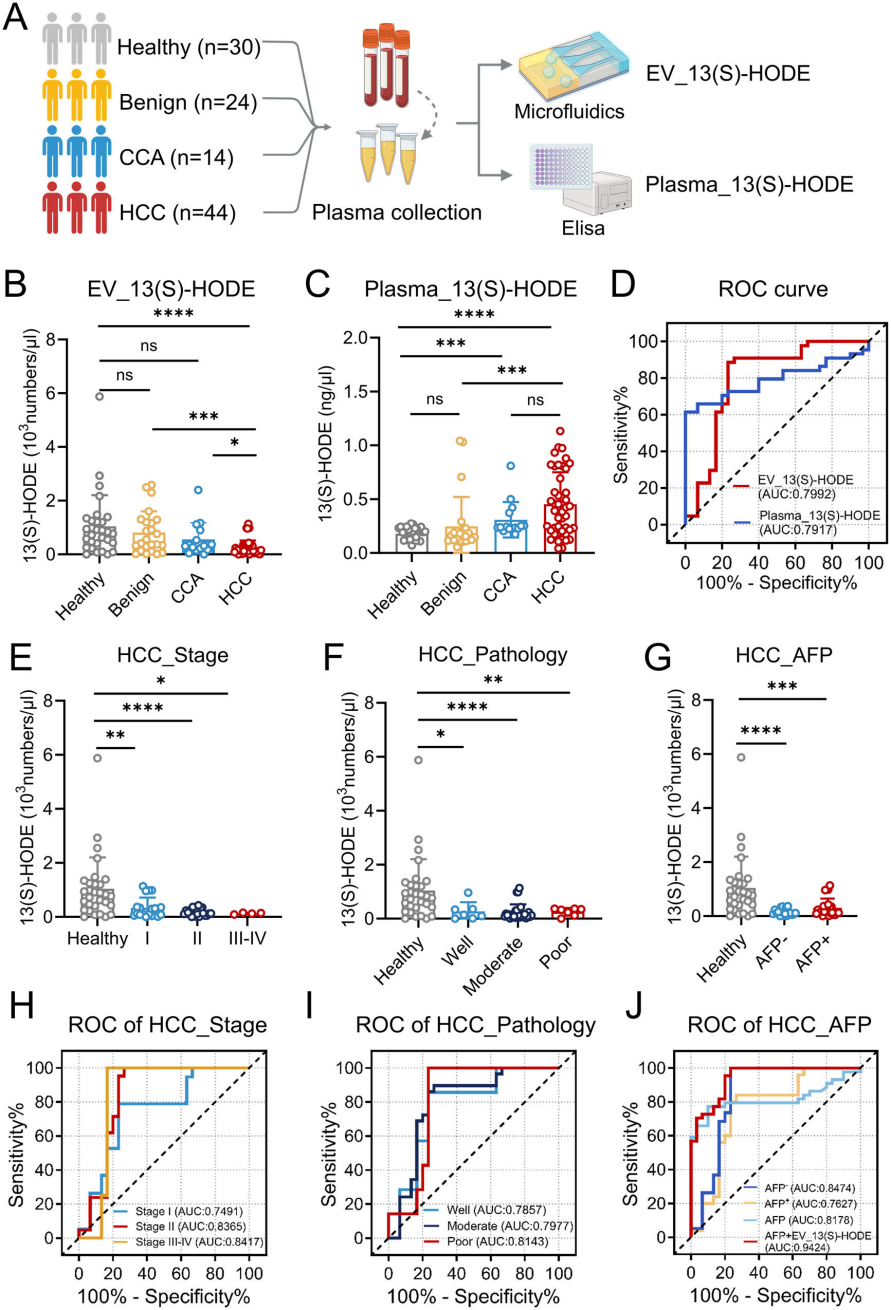
The diagnostic performance of plasma derived 13(S)‐HODE‐positive EVs in HCC. (A) Schematic diagram of the workflow for detecting total plasma 13(S)‐HODE (Plasma_13(S)‐HODE) and plasma EV‐derived 13(S)‐HODE (EV_13(S)‐HODE). (B‐C) Comparison of EV_13(S)‐HODE and Plasma_13(S)‐HODE levels among four groups: Healthy control group (Healthy, *n* = 30), Benign hepatobiliary disease group (Benign, *n* = 24), Cholangiocarcinoma group (CCA, *n* = 15), and Hepatocellular carcinoma group (HCC, *n* = 44). Mann‐Whitney test, *p < 0.05, ***p < 0.001, ****p < 0.0001. (D) ROC curve comparison between EV_13(S)‐HODE and Plasma_13(S)‐HODE for HCC diagnosis. (E‐G) Comparison of EV_13(S)‐HODE levels in different subgroups of HCC patients: (E) TNM stages: Stage I (*n* = 19), Stage II (*n* = 21), Stage III‐IV (*n* = 4). (F) Tumor differentiation: Poor (*n* = 7), Moderate (*n* = 19), Well (*n* = 7). (G) Serum AFP status: Negative (*n* = 19), Positive (*n* = 25). Mann‐Whitney test, *p < 0.05, **p < 0.01, ***p < 0.001, ****p < 0.0001. (H‐J) ROC curves of EV_13(S)‐HODE for diagnosing HCC in various subgroups: (H) Stratified by TNM stage. (I) Stratified by differentiation grade. (J) Stratified by AFP status (AFP‐negative AUC = 0.8474, AFP‐positive AUC = 0.7627), along with comparison of the diagnostic efficacy of AFP alone (AUC = 0.8178) and AFP combined with EV_13(S)‐HODE (AUC = 0.9424). Samples of healthy donors for detection of EV‐13S‐HODE in B, E‐G, derived from the same group.

Previous studies indicated that EV cargo can be quantified in both tissue and plasma, suggesting that tissue derived EVs may enter the bloodstream and finally reflect the status of the tumour (Hoshino et al. 2020). Therefore, to further evaluate whether 13(S)‐HODE level has the same performance between plasma and tissue, the droplet microfluidics‐based ddPCR platform were used to quantify its level across four distinct clinical cohorts, including healthy controls (healthy, *n* = 30), patients with benign hepatobiliary disease (benign, *n* = 24), cholangiocarcinoma (CCA, *n* = 15), and hepatocellular carcinoma (HCC, *n* = 44) (Figure 5A). The results showed that compared with healthy controls, EV 13(S)‐HODE levels were significantly reduced only in HCC patients (*p* < 0.0001), whereas no statistically significant differences were observed in the benign (*p* = 0.4214) or CCA groups (*p* = 0.0615), suggesting that the downregulation of EV 13(S)‐HODE is specific to HCC (Figure 5B). Further analysis indicated that EV 13(S)‐HODE levels in the HCC group were also significantly lower than those in the benign (*p* = 0.0008) and CCA (*p* = 0.0253) groups, highlighting its potential for differential diagnosis of HCC (Figure 5B). Intriguingly, results from ELISA showed opposite trends between the total plasma pool and the EV sub‐pool: total plasma 13(S)‐HODE levels were significantly elevated in HCC patients (Figure 5C), while EV 13(S)‐HODE levels were significantly reduced (Figure 5B). This dissociation—elevated total levels but reduced EV levels—suggests potential dysregulation of lipid sorting and packaging during HCC progression. Specifically, 13(S)‐HODE may be preferentially released into the bloodstream in its free form rather than actively exported via EVs in the tumor state. Notably, the downregulation of EV 13(S)‐HODE observed in plasma was consistent with the trend previously identified in tumor tissue‐derived EVs (Figure 3A), reinforcing that plasma EV 13(S)‐HODE may better reflect the active secretory status of cells within the tumor microenvironment—an insight unattainable from total plasma levels alone. Despite the opposing trends, ROC curve analysis revealed that EV‐derived 13(S)‐HODE showed slightly superior diagnostic performance compared to total plasma 13(S)‐HODE (AUC = 0.7992 vs. 0.7917, respectively. Figure 5D). To further evaluate the diagnostic value of plasma EV‐derived 13(S)‐HODE across HCC subtypes, we analysis subtype based on key clinicopathological characteristics, including TNM stage, tumor grade, and serum AFP level. These results indicated that plasma EV‐derived 13(S)‐HODE levels showed significant alterations across all HCC subtypes analyzed (Figure 5E–G) and consistently demonstrated favorable diagnostic performance (AUC > 0.70) within each subgroup (Figure 5H–J), while plasma 13(S)‐HODE do not differ significantly among patient cohorts, even though in the frozen samples, like Figure S2C (Figure S2). The qualification of plasma EV‐derived 13(S)‐HODE level exhibited superior performance in intermediate–advanced stages (stages II–IV, AUC = 0.8417) and in poorly/moderately differentiated tumors (AUC > 0.80) (Figure 5H–J). Notably, its diagnostic efficacy was significantly higher in AFP‐negative patients than in AFP‐positive patients (AUC = 0.8474 vs. 0.7627. Figure 5J), suggesting that EV 13(S)‐HODE may have greater sensitivity for detecting AFP‐negative HCC (cut‐off: 13(S)‐HODE < 390.3 numbers/µl, sensitivity = 100.0%, specificity = 76.67%), which is often clinically challenging. We further assessed the diagnostic value of combining EV 13(S)‐HODE with AFP using ROC curve analysis. In this cohort, AFP alone yielded an AUC of 0.8178 for diagnosing HCC. Notably, combining EV 13(S)‐HODE with AFP significantly enhanced diagnostic accuracy, achieving an AUC of 0.9424 (Figure 5J). Above all, these data validating plasma EV‐derived 13(S)‐HODE as a complementary biomarker for HCC with high diagnostic accuracy across diverse subtypes and AFP‐negative patients.

### 3.5 Clinical Validation of 13(S)‐HODE‐EVs Based Machine Learning Model for HCC Prediction

**FIGURE 6:**
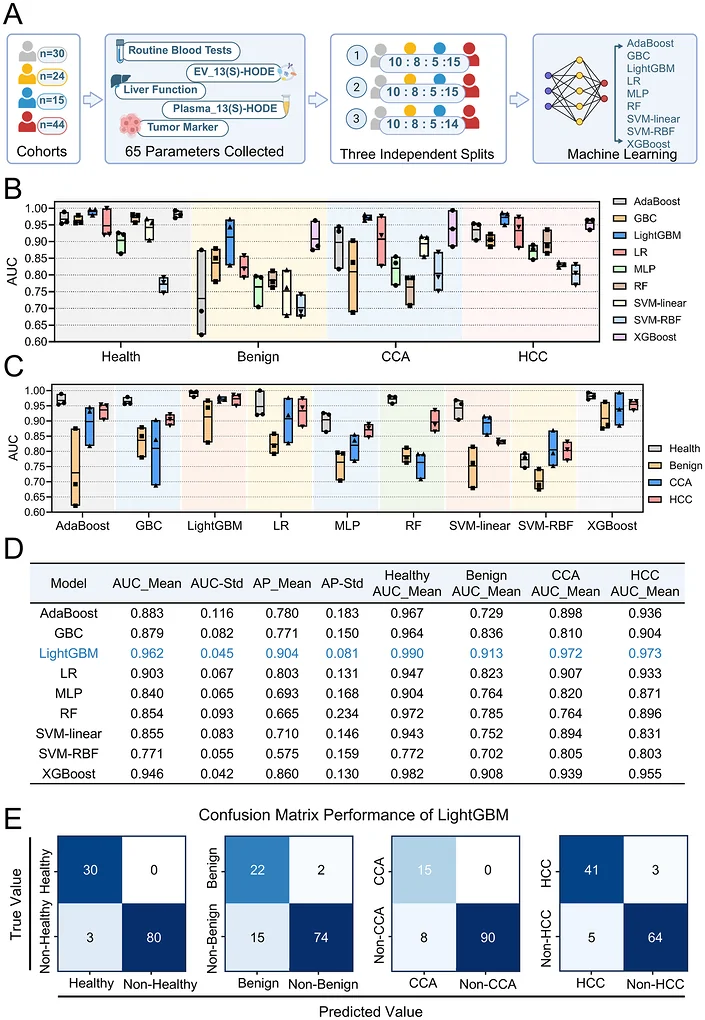
Clinical validation of 13(S)‐HODE‐EVs based machine learning model for HCC prediction. (A) Workflow for model construction and evaluation. (B) Performance (AUC) of nine algorithms in discriminating four cohorts across three validation splits by using training and validation cohort. (C) Comparison of overall four‐group classification performance (AUC) across algorithms. (D) Summary table of average performance metrics for each algorithm. (E) Confusion matrix for the optimal LightGBM model.

To further improve the diagnostic accuracy of HCC, we integrated the 13(S)‐HODE levels and corresponding clinical parameters (including blood routine, liver function, classic tumor biomarkers, etc., Table S4) from the same clinical cohort of 113 individuals preciously used for 13(S)‐HODE measurement. Nine machine learning algorithms were employed to construct a multi‐class diagnostic model, including adaptive boosting (Ada), gradient boosting classifier (Gbc), light gradient boosting machine (LightGBM), logistic regression (LR), multilayer perceptron (MLP), random forest (RF), support vector machine linear (SVM‐linear), radial basis function support vector machine (SVM‐RBF), and extreme gradient boosting (XGBoost). We first completed full hyperparameter tuning independently prior to carrying out three‐fold cross‐validation, hyperparameters were fixed throughout all folds of cross‐validation. Model performance was assessed using metrics such as accuracy, area under the curve (AUC) of the ROC curve, sensitivity, specificity, precision, as described in Figure 6A. Results demonstrated that all algorithms achieved robust performance in discriminating between healthy controls and HCC patients (AUC > 0.75 across all three validation splits) (Figure 6B). After evaluating various models, the LightGBM algorithm was selected for its superior performance (Figure 6C), exhibiting outstanding discriminatory power for each group (AUC > 0.90) (Figure 6D). The confusion matrix of the LightGBM model further confirmed its excellent diagnostic ability (Figure 6E). Moreover, EV_13‐HODE was among the top 15 most important parameters in the LightGBM model (Table S5), suggesting that 13(S)‐HODE holds significant value in the diagnosis of HCC. Furthermore, we have further evaluated the diagnostic performance of combinations of several high‐ranking factors identified by LightGBM modeling, together with EV‐13S‐HODE in our cohort. The results showed that 13S‐HODE‐EVs addition with other panels remarkably improved the diagnostic performance for HCC, both in biomarker sensitivity and specificity (Table S6). In summary, we developed a high‐performance diagnostic model based on LightGBM, underscoring the potential of combining large scale clinical data to achieve more comprehensive and reliable HCC screening in the future.

## 4 Discussion

Current HCC blood biomarkers such as AFP and PIVKA‐II, have limitations in accuracy (Donne and Lujambio 2023; Greten et al. 2023; Ricco et al. 2020; Tayob et al. 2023). EVs, carrying disease‐specific signals, offer a promising alternative (Casanova‐Salas et al. 2024; Crescitelli et al. 2025; Hoshino et al. 2020; Shah et al. 2018; Théry et al. 2018; Welsh et al. 2024). Our integrated analysis of plasma and tissue EVs led to an oxylipin panel 13(S)‐HODE. Moreover, we established a translational microfluidic assay with single‐molecule resolution to detect 13(S)‐HODE in plasma EVs. The machine learning model LightGBM based on 13(S)‐HODE and conventional clinical data (blood routine, liver function and classic tumor biomarkers) exhibit outstanding diagnostic performance for HCC.

In our study, we employed advanced oxylipinomic technologies to comprehensively profile EV oxylipin derived from HCC tissues and adjacent non‐tumor tissues. Our results revealed significant downregulation of 10 oxylipins—including the hub oxlipin 13(S)‐HODE—in tumour EVs, while oxylipin contents were not distinctly different in the solid tissues (the adjacent and the core), demonstrating that tissue‐derived EVs were more abundant and carried distinct oxlipin profiles compared to solid tissue counterparts. This suggests that tissue derived EVs harbor a richer reservoir of molecular information of tumor. Functional analysis showed that the tissue EV oxlipins could reflect the metabolism features of cancer, especially the linoleic acid oxidation metabolism. Notably, our results revealed that 13(S)‐HODE of EV particles in HCC patients from plasma significantly exhibited conformity with tissue, demonstrating that screening EV oxlipins shared between both tissue and plasma enhances the likelihood of discovering tumor‐specific biomarkers. This integrated strategy holds great potential for the future discovery of diagnostic biomarkers. Moreover, in contrast to its significant reduction within plasma‐derived EVs, 13(S)‐HODE was increased in the free state within HCC patient plasma. This observation suggests that oxylipins may be metabolized or sorted differently between EVs and the free form. We hypothesize that under physiological conditions, oxylipins are selectively sorted into EV membranes or lumens to maintain their stability and participate in intercellular communication, whereas in HCC, the EV oxylipin sorting machinery may become dysregulated. This could lead to inefficient packaging of oxylipins into EVs, resulting in their release into circulation in free form and a concomitant decrease in EV‐associated oxylipins. This hypothesis aligns with previous findings that oxylipins can be anchored to EV membranes via phospholipid esters or encapsulated within EVs to avoid degradation (Biagini et al. 2024; Pizzinat et al. 2020). Previous studies reported that, oxylipin analysis of serum samples from HBV‐related HCC patients demonstrated that 13‐HODE, along with linoleic acid (LA) and 9‐HODE, exhibited potential in distinguishing HCC patients from healthy controls. Mechanistically, 13(S)‐HODE is a product of 15‐lipoxygenase (15‐LOX)‐mediated metabolism of linoleic acid and functions as an endogenous ligand for peroxisome proliferator‐activated receptor gamma (PPARγ^)^ (Ning et al. 2022). It also has been shown to inhibit tumor cell adhesion in various biological systems. Besides, early in vivo studies provided evidence that 13S‐HODE serves as a mitogenic signal for linoleic acid‐dependent tumor growth and response to growth factors or regulate apoptosis in human colon cancers (Eling and Glasgow 1994; Shureiqi et al. 1999; Shureiqi et al. 2003). However, the significance of signal transduction and future studies are needed to elucidate the key molecular mechanisms regulating EV sorting of oxylipins and their role in HCC development.

Given the extremely low abundance of EVs and the complex background of circulating biomolecules in plasma, traditional detection methods are insufficient for achieving high‐sensitivity, quantitative analysis. Building on our group's previously developed single‐EV protein and mRNA detection platforms (Liu et al. 2018; Liu et al. 2023; Liu et al. 2021), we have further established a single‐molecule quantitative detection system for EV membrane oxylipins. This platform integrates antibody‐oligonucleotide conjugation technology with microfluidic droplet digital PCR to achieve absolute quantification of 13(S)‐HODE on plasma EVs at single‐molecule sensitivity, effectively overcoming interference from the complex plasma background. This provides reliable technical support for the clinical translation of EV oxylipin biomarkers. The development of such technology represents an important direction in the evolution of liquid biopsy from biomarker discovery toward precise quantitative diagnosis, with broad prospects for early HCC screening and treatment monitoring. Furthermore, when lysed EVs/total EVs were treated with /without 0.1% Triton X‐100 (helping to release EV cargoes), Elisa detection showed an enhanced 13(S)‐Hode level in the 0.1% Triton X‐100 treated group compared with total EVs (Figure S3). These results suggests that 13(S)‐HODE may partially encapsulated in the vesicles, rather than just merely on the EV surface. Furthermore, the stability of EV‐13(S)‐HODE against enzymatic degradation is likely attributable to the protective lipid bilayer of EVs, as also illustrated in the previous study (Wu et al. 2025). Although this does not affect the capture of 13S‐HODE positive EVs by using microfluidic, the intra‐vesicular cargo study also has critical impact on the understanding about EV biogenesis and cargo packaging. Therefore, the mechanism of the cargo selection and metabolic signal transduction needs to be further clarified in the future.

In terms of clinical translation, our study not only validates the independent diagnostic value of EV 13(S)‐HODE in HCC but also integrates multidimensional clinical data—including routine blood tests, liver function indicators, and traditional tumor markers such as AFP—to construct a machine learning‐based integrated diagnostic model using LightGBM. The model demonstrated excellent discriminatory performance (AUC > 0.90) in four cohorts (healthy donors, benign observations, CCA and HCC patients), significantly improving overall diagnostic accuracy and robustness. This multimodal integration strategy—combining EV biomarkers with routine clinical parameters—not only enhances clinical applicability but also offers a new approach to personalized medicine.

Limitations of the present study should also be acknowledged. Firstly, the initial oxylipin‐omics discovery was conducted on a relatively small cohort of seven matched pairs of HCC and adjacent non‐tumor tissues. Tumor tissues exhibited considerable inter‐ and intra‐tumoral heterogeneity (Fan et al. 2025), which is further driven by diverse backgrounds (including HBV, HCV, NAFLD, and alcohol‐related liver disease), varying tumor stages, and distinct molecular subtypes. Consequently, such a limited discovery cohort may carry an inherent risk of false‐positive biomarker identification and selection bias. The independent matched‐pair design replication should be adopted to partially mitigate inter‐individual variability in the future. Secondly, although the original discovery of a 13S‐HODE‐enriched subpopulation as a promising diagnostic biomarker for HCC by using oxylipidomics platform and single‐molecule technology, the current findings are based on a single‐center cohort, which limits the clinical applications. Therefore, rigorous multi‐center validation is critical before this candidate biomarker can be translated into routine clinical practice. A comprehensive evaluation of the robustness, reproducibility, and clinical value of the 13S‐HODE‐associated signature is ongoing. Besides, emerging evidence also indicates that EVs derived from highly metastatic colorectal cancer cells drive hepatic lipid accumulation and premetastatic niche formation through the upregulation of fatty acid synthesis and the deliver proteinogenic lipids (Cao et al. 2025). Although speculative, lipidomic evidence suggests that 15‐LOX‐1 and its product 13(S)‐HODE are downregulated in colorectal cancer and inversely associated with metastatic potential (Liu et al. 2020), raising the possibility that EV_13(S)‐HODE levels may differ between HCC and colorectal liver metastasis (CRLM). Future studies comparing the EV signature difference between CRLM and HCC samples are essential to further validate the biomarker's performance.

In conclusion, this study reveals the potential of EV‐associated oxylipin 13(S)‐HODE as a novel diagnostic biomarker for HCC, suggests that its differential expression between EV and free forms may involve reprogramming of lipid sorting mechanisms, develops a single‐molecule detection platform suitable for EV lipid analysis, and constructs a high‐performance diagnostic model through integration of clinical data. These findings provide new perspectives, methods, and tools for early HCC diagnosis and lay the groundwork for further exploration of EV lipid biology and its regulatory mechanisms in liver cancer.

## Author Contributions

**Zhenxun Wang**: methodology. **Qiaoting Wu**: writing – original draft, validation, software. **Yingxue Shen**: validation. **Rong Wang**: methodology, software. **Yu Wang**: supervision, project administration, funding acquisition. **Xin Zhang**: writing – review and editing, writing – original draft, supervision, funding acquisition, conceptualization, investigation. **Wanyang Sun**: resources. **Xiaoqing Jiang**: data curation. **Chao Yang**: methodology, software. **Rongrong He**: resources. **Lei Zheng**: supervision, project administration, funding acquisition, resources. **Jiaming Chen**: validation. **Huixian Lin**: methodology, writing – original draft. **Chunchen Liu**: funding acquisition, writing – original draft, methodology, resources. **Bo Ma**: validation. **Jiawei Li**: validation.

## Funding

This work was supported by National Key Research and Development Program (#2024YFC2419102), National Natural Science Foundation of China (#82172966, #82230080, #82372346), Basic and Applied Basic Research Project of Guangdong Province (#2023B1515230007, #2024A1515010382, #2023A1515220091), Science and Technology Program of Guangzhou (#2025B03J0116), and Outstanding Youths Development Scheme of Nanfang Hospital, Southern Medical University (#2025J011), the Sichuan Provincial Natural Science Foundation Project (2024NSFSC1897), Jiangxi Provincial Natural Science Foundation (20262BAC220049), Guang Dong Basic and Applied Basic Research Foundation (2025A1515220122).

## Ethics Statement

All procedures performed in this study with human biological specimens were in accordance with the ethical standards established by the Ethics Committee of Nanfang Hospital in Southern Medical University (Ethics approval number: NFEC‐2022‐056) This study was conducted in accordance with the principles of the Declaration of Helsinki. All clinical samples used in this study were obtained with informed consent from the patients.

## Conflicts of Interest

The authors declare no conflicts of interest.

## Supporting information




**Supporting Information**: jev270368‐sup‐0001‐SuppMat.docx

## Data Availability

The datasets generated during and analyzed in the study are available from the corresponding author for reasonable request. The normalized oxylipin profiling datasets have been deposited in the OMIX, China National Center for Bioinformation/Beijing Institute of Genomics, Chinese Academy of Sciences (accession number: OMIX015519, https://ngdc.cncb.ac.cn/omix/preview/ff3ruP7Z).

## References

[jev270368-bib-0001] Biagini, D. , S. Mrakic‐Sposta , D. Bondi , et al. 2024. “A MEPS‐UHPLC‐MS/MS Analytical Platform to Detect Isoprostanoids and Specialized Pro‐Resolving Mediators in the Urinary Extracellular Vesicles of Mountain Ultramarathon Runners.” Talanta 279: 126619. 10.1016/j.talanta.2024.126619 39067203

[jev270368-bib-0002] Cabral, M. , R. Martín‐Venegas , and J. J. Moreno . 2014. “Differential Cell Growth/Apoptosis Behavior of 13‐Hydroxyoctadecadienoic Acid Enantiomers in a Colorectal Cancer Cell Line.” American Journal of Physiology‐Gastrointestinal and Liver Physiology 307, no. 6: G664–G671. 10.1152/ajpgi.00064.2014 25035111

[jev270368-bib-0003] Cao, J. , S. Qin , B. Li , et al. 2025. “Extracellular Vesicle‐Induced Lipid Dysregulation Drives Liver Premetastatic Niche Formation in Colorectal Cancer.” Gut 74, no. 12: 2012–2023. 10.1136/gutjnl-2025-334851 40562522

[jev270368-bib-0004] Casanova‐Salas, I. , D. Aguilar , S. Cordoba‐Terreros , et al. 2024. “Circulating Tumor Extracellular Vesicles to Monitor Metastatic Prostate Cancer Genomics and Transcriptomic Evolution.” Cancer Cell 42, no. 7: 1301–1312.e7. 10.1016/j.ccell.2024.06.003 38981440

[jev270368-bib-0005] Chistyakov, D. V. , L. V. Kovalenko , M. Y. Donnikov , and M. G. Sergeeva . 2023. “Blood Oxylipin Profiles as Markers of Oncological Diseases.” Biochemistry (Moscow) 88, no. 5: 621–629. 10.1134/S000629792305005X 37331708

[jev270368-bib-0006] Crescitelli, R. , Y. Huang , A. Hendrix , et al. 2025. “Recommendations for Studying in Situ Extracellular Vesicles From Solid Tissue.” Journal of Extracellular Vesicles 14, no. 11: e70185. 10.1002/jev2.70185 PMC12606037 41220186

[jev270368-bib-0007] Donne, R. , and A. Lujambio . 2023. “The Liver Cancer Immune Microenvironment: Therapeutic Implications for Hepatocellular Carcinoma.” Hepatology 77, no. 5: 1773–1796. 10.1002/hep.32740 PMC9941399 35989535

[jev270368-bib-0008] Eling, T. E. , and W. C. Glasgow . 1994. “Cellular Proliferation and Lipid Metabolism: Importance of Lipoxygenases in Modulating Epidermal Growth Factor‐Dependent Mitogenesis.” Cancer and Metastasis Reviews 13, no. 3‐4: 397–410. 10.1007/BF00666106 7712598

[jev270368-bib-0009] Fan, B. , J. Zhang , C. Li , et al. 2025. “New Trend in Molecular Diagnostics: Insights From the Tumor Microenvironment.” Interdisciplinary Medicine 3: e70048.

[jev270368-bib-0010] Greten, T. F. , A. Villanueva , F. Korangy , et al. 2023. “Biomarkers for Immunotherapy of Hepatocellular Carcinoma.” Nature Reviews Clinical Oncology 20, no. 11: 780–798. 10.1038/s41571-023-00816-4 37726418

[jev270368-bib-0011] Guri, Y. , M. Colombi , E. Dazert , et al. 2017. “mTORC2 Promotes Tumorigenesis via Lipid Synthesis.” Cancer Cell 32, no. 6: 807–823.e12. 10.1016/j.ccell.2017.11.011 29232555

[jev270368-bib-0012] Hateley, C. , A. Olona , L. Halliday , et al. 2024. “Multi‐Tissue Profiling of Oxylipins Reveal a Conserved up‐Regulation of Epoxide:Diol Ratio That Associates With White Adipose Tissue Inflammation and Liver Steatosis in Obesity.” EBioMedicine 103: 105127. 10.1016/j.ebiom.2024.105127 PMC11061246 38677183

[jev270368-bib-0013] Hoshino, A. , H. S. Kim , L. Bojmar , et al. 2020. “Extracellular Vesicle and Particle Biomarkers Define Multiple Human Cancers.” Cell 182, no. 4: 1044–1061.e18. 10.1016/j.cell.2020.07.009 PMC7522766 32795414

[jev270368-bib-0014] Il Lee, S. , X. Zuo , and I. Shureiqi . 2011. “15‐Lipoxygenase‐1 as a Tumor Suppressor Gene in Colon Cancer: Is the Verdict in?” Cancer and Metastasis Reviews 30, no. 3‐4: 481–491. 10.1007/s10555-011-9321-0 PMC3261081 22037943

[jev270368-bib-0015] Lehrich, B. M. , J. Zhang , S. P. Monga , and R. Dhanasekaran . 2024. “Battle of the Biopsies: Role of Tissue and Liquid Biopsy in Hepatocellular Carcinoma.” Journal of Hepatology 80, no. 3: 515–530. 10.1016/j.jhep.2023.11.030 PMC10923008 38104635

[jev270368-bib-0016] Liu, C. , B. O. Li , H. Lin , et al. 2021. “Multiplexed Analysis of Small Extracellular Vesicle‐Derived mRNAs by Droplet Digital PCR and Machine Learning Improves Breast Cancer Diagnosis.” Biosensors and Bioelectronics 194: 113615. 10.1016/j.bios.2021.113615 34507095

[jev270368-bib-0017] Liu, C. , H. Lin , J. Guo , et al. 2023. “Profiling of Single‐Vesicle Surface Proteins via Droplet Digital Immuno‐PCR for Multi‐Subpopulation Extracellular Vesicles Counting Towards Cancer Diagnostics.” Chemical Engineering Journal 471: 144364.

[jev270368-bib-0018] Liu, C. , X. Xu , B. Li , et al. 2018. “Single‐Exosome‐Counting Immunoassays for Cancer Diagnostics.” Nano Letters 18, no. 7: 4226–4232. 10.1021/acs.nanolett.8b01184 29888919

[jev270368-bib-0019] Liu, F. , X. Zuo , Y. I. Liu , et al. 2020. “Suppression of Membranous LRP5 Recycling, Wnt/β‐Catenin Signaling, and Colon Carcinogenesis by 15‐LOX‐1 Peroxidation of Linoleic Acid in PI3P.” Cell Reports 32, no. 7: 108049. 10.1016/j.celrep.2020.108049 PMC8765570 32814052

[jev270368-bib-0020] Liu, H. , T. Cheng , G. Li , et al. 2025. “Organoid‐Tissue Extracellular Vesicles.” Interdisciplinary Medicine 3, no. 5.

[jev270368-bib-0021] Liu, Q. , X. Zhang , J. Qi , et al. 2025. “Comprehensive Profiling of Lipid Metabolic Reprogramming Expands Precision Medicine for HCC.” Hepatology 81, no. 4: 1164–1180. 10.1097/HEP.0000000000000962 PMC11902616 38899975

[jev270368-bib-0022] Ning, Z. , X. Guo , X. Liu , et al. 2022. “USP22 Regulates Lipidome Accumulation by Stabilizing PPARγ in Hepatocellular Carcinoma.” Nature Communications 13, no. 1: 2187. 10.1038/s41467-022-29846-9 PMC9023467 35449157

[jev270368-bib-0023] Pizzinat, N. , V. Ong‐Meang , F. Bourgailh‐Tortosa , et al.: 2020. “Extracellular Vesicles of MSCs and Cardiomyoblasts are Vehicles for Lipid Mediators.” Biochimie 178: 69–80. 10.1016/j.biochi.2020.07.013 32835733

[jev270368-bib-0024] Ricco, G. , C. Cosma , G. Bedogni , et al. 2020. “Modeling the Time‐Related Fluctuations of AFP and PIVKA‐II Serum Levels in Patients With Cirrhosis Undergoing Surveillance for Hepatocellular Carcinoma.” Cancer Biomarkers 29, no. 2: 189–196. 10.3233/CBM-190118 PMC12662534 32623383

[jev270368-bib-0025] Shah, R. , T. Patel , and J. E. Freedman . 2018. “Circulating Extracellular Vesicles in Human Disease.” New England Journal of Medicine 379, no. 22: 2180–2181. 10.1056/NEJMc1813170 30485772

[jev270368-bib-0026] Shin, M. , and Y. Byun . 2024. “Stereoselective Syntheses of Polyunsaturated Fatty Acids, 13‐( S )‐HODE and 15‐( S )‐HETE.” Journal of Organic Chemistry 89, no. 16: 11293–11303. 10.1021/acs.joc.4c00983 39096279

[jev270368-bib-0027] Shureiqi, I. , W. Jiang , X. Zuo , et al. 2003. “The 15‐Lipoxygenase‐1 Product 13‐ S ‐Hydroxyoctadecadienoic Acid Down‐Regulates PPAR‐δ to Induce Apoptosis in Colorectal Cancer Cells.” Proceedings of the National Academy of Sciences 100, no. 17: 9968–9973. 10.1073/pnas.1631086100 PMC187904 12909723

[jev270368-bib-0028] Shureiqi, I. , K. J. Wojno , J. A. Poore , et al. 1999. “Decreased 13‐S‐Hydroxyoctadecadienoic Acid Levels and 15‐Lipoxygenase‐1 Expression in Human Colon Cancers.” Carcinogenesis 20, no. 10: 1985–1995. 10.1093/carcin/20.10.1985 10506115

[jev270368-bib-0029] Siegel, R. L. , T. B. Kratzer , N. S. Wagle , H. Sung , and A. Jemal . 2026. “Cancer Statistics, 2026.” CA: A Cancer Journal for Clinicians 76, no. 1: e70043. 10.3322/caac.70043 PMC12798275 41528114

[jev270368-bib-0030] Surmiak, M. , A. Gielicz , D. Stojkov , et al. 2020. “LTB4 and 5‐oxo‐ETE From Extracellular Vesicles Stimulate Neutrophils in Granulomatosis With Polyangiitis.” Journal of Lipid Research 61, no. 1: 1–9. 10.1194/jlr.M092072 PMC6939603 31740445

[jev270368-bib-0031] Tayob, N. , F. Kanwal , A. Alsarraj , R. Hernaez , and H. B. El‐Serag . 2023. “The Performance of AFP, AFP‐3, DCP as Biomarkers for Detection of Hepatocellular Carcinoma (HCC): A Phase 3 Biomarker Study in the United States.” Clinical Gastroenterology and Hepatology 21, no. 2: 415–423.e414. 10.1016/j.cgh.2022.01.047 PMC9346092 35124267

[jev270368-bib-0032] Théry, C. , K. W. Witwer , E. Aikawa , et al. 2018. “Minimal Information for Studies of Extracellular Vesicles 2018 (MISEV2018): A Position Statement of the International Society for Extracellular Vesicles and Update of the MISEV2014 Guidelines.” Journal of Extracellular Vesicles 7, no. 1: 1535750. 10.1080/20013078.2018.1535750 PMC6322352 30637094

[jev270368-bib-0033] Wang, X. , L. Zhang , and B. Dong . 2025. “Molecular Mechanisms in MASLD/MASH‐Related HCC.” Hepatology 82, no. 5: 1303–1324. 10.1097/HEP.0000000000000786 PMC11323288 38349726

[jev270368-bib-0034] Welsh, J. A. , D. C. I. Goberdhan , L. O'Driscoll , et al. 2024. “Minimal Information for Studies of Extracellular Vesicles (MISEV2023): From Basic to Advanced Approaches.” Journal of Extracellular Vesicles 13, no. 2: e12404. 10.1002/jev2.12404 PMC10850029 38326288

[jev270368-bib-0035] Wu, B. , Z. Wang , G. Wang , et al. 2025. “Quantification of Fascin‐1‐Positive Extracellular Vesicles by Nanoflow Cytometry for Early Detection of Hepatocellular Carcinoma in Liquid Biopsy.” International Journal of Medical Sciences 22, no. 7: 1574–1584. 10.7150/ijms.102438 PMC11905273 40093808

[jev270368-bib-0036] Yuan, H. , M. Y. Li , L. T. Ma , et al. 2010. “15‐Lipoxygenases and Its Metabolites 15(S)‐HETE and 13(S)‐HODE in the Development of Non‐Small Cell Lung Cancer.” Thorax 65, no. 4: 321–326. 10.1136/thx.2009.122747 20388757

[jev270368-bib-0037] Zhang, X. , J. Li , Y. Huang , et al. 2025. “Plasma Extracellular Vesicles From Recurrent GBMs Carrying LDHA to Activate Glioblastoma Stemness by Enhancing Glycolysis.” Theranostics 15, no. 8: 3655–3672. 10.7150/thno.102014 PMC11905145 40093910

[jev270368-bib-0038] Zhang, X. , Y. I. Liu , L. Dai , et al. 2021. “BATF2 prevents Glioblastoma Multiforme Progression by Inhibiting Recruitment of Myeloid‐Derived Suppressor Cells.” Oncogene 40, no. 8: 1516–1530. 10.1038/s41388-020-01627-y PMC7906906 33452462

[jev270368-bib-0039] Zhu, Y. , W. Li , F. Lan , et al.: 2023. “DNA Nanotechnology in Tumor Liquid Biopsy: Enrichment and Determination of Circulating Biomarkers.” Interdisciplinary Medicine 2, no. 1: e20230043.

