# Supplementary material for: Oxylipins‐Omics Combine With Single‐Molecule Analysis Identifies 13(S)‐HODE Positive Extracellular Vesicles as a Diagnostic Biomarker for AFP‐Negative Hepatocellular Carcinoma
Source: J Extracell Vesicles. 2026 Sep 17;15(9):e70368. doi: 10.1002/jev2.70368 (PMC13586547; doi:10.1002/jev2.70368)
Supplement: Supplementary file 1 [file JEV2-15-e70368-s001.docx]

**Supplementary Figure 1:**

**Supplementary Figure 1**: Nano-FCM analysis and statistics of CD63 positive EVs in

Blank control (A) and plasma derived EVs (B).

**Supplementary Figure 2.**

**Supplementary Figure 2.** Comparison of Plasma_13(S)-HODE levels in different subgroups of HCC patients: (A) TNM stages: Stage I (n=17), Stage II (n=15), Stage III-IV (n=11). (B) Tumor differentiation: Poor (n=8), Moderate (n=10), Well (n=10). (C) Serum AFP status: Negative (n=19), Positive (n=25). Healthy, (A), n=20. (B), n=14. (C), n=30. Mann-Whitney test, ns. Non-significant. **p* < 0.05, ***p* < 0.01.

**Supplementary Figure 3:** ELISA detection of 13(S)-HODE with and without 0.1% Triton X-100 in tissue derived EVs (TD-EVs) and plasma derived EVs (P-EVs) (n= 6, independent experiment, ***p*< 0.01).

**Table S1. Inclusion and Exclusion Criteria of the four study cohorts**

**Table S1-1. Patients with hepatocellular carcinoma (HCC, n=44)**

**Inclusion criteria**

(1) Patients with a first diagnosis of HCC who underwent liver resection.
(2) Histologically confirmed HCC tissues.
(3) Availability of complete key clinical data, including complete blood count, liver function tests and classic tumor biomarkers (e.g., CA19-9, CEA, AFP).

**Exclusion criteria**

(1) patients with recurrent HCC.
(2) undergoing preoperative loco-regional or systemic therapies or adjuvant treatments.
(3) Insufficient data or data not available.
(4) presence of other primary malignancies.
(5) mixed histology of cholangiocellular carcinoma and HCC.

**Table S1-2. Patients with cholangiocarcinoma (CCA, n=15)**

**Inclusion criteria**

(1) Patients with a first diagnosis of CCA who underwent liver resection.
(2) Histologically confirmed CCA tissues.
(3) Availability of complete key clinical data, including complete blood count, liver function tests and classic tumor biomarkers (e.g., CA19-9, CEA, AFP).

**Exclusion criteria**

(1) patients with recurrent CCA.
(2) undergoing preoperative loco-regional or systemic therapies or adjuvant treatments.
(3) Insufficient data or data not available.
(4) presence of other primary malignancies.
(5) mixed histology of cholangiocellular carcinoma and HCC .

**Table S1-3. Patients with benign hepatobiliary disease (benign, n=24)**

**Inclusion Criteria**

(1) Patients with a confirmed benign hepatobiliary lesion, diagnosed either by pathology or characteristic imaging findings.
(2) Availability of complete key clinical data, including complete blood count, liver function tests and classic tumor biomarkers (e.g., CA19-9, CEA, AFP).

**Exclusion Criteria**

(1) Presence or history of any malignancy.
(2) Prior specific intervention (e.g., resection, ablation) for the benign lesion before baseline data and blood collection.
(3) Insufficient data or data not available.

**Table S1-4. Healthy controls (healthy, n=30)**

**Inclusion criteria**

(1) Individuals who underwent a comprehensive health check-up at Nanfang Hospital with all results assessed as normal.
(2) Normal liver function tests and abdominal ultrasound.
(3) Availability of complete key clinical data, including complete blood count, liver function tests and classic tumor biomarkers (e.g., CA19-9, CEA, AFP).

**Exclusion Criteria**

(1) History of any hepatobiliary disease, chronic illness, or malignancy.
(2) Abnormal serology (e.g., positive HBsAg), elevated tumor markers (e.g., AFP, CA19-9), or other clinically significant lab abnormalities.
(3) History of excessive alcohol consumption, acute illness within one month, pregnancy/lactation, or use of hepatotoxic medications.

**Table S1-5. Summary of Benign Hepatobiliary Lesions**

| **Disease Category** | **Specific Diagnoses (Number of Cases)** | **Total Cases** |
| --- | --- | --- |
| Gallbladder Inflammatory Diseases | Cholecystolithiasis with Chronic Cholecystitis (2), Acute Suppurative Cholecystitis (1), Chronic Cholecystitis with Cholesterol Polyp (3), Chronic Cholecystitis (1) | 7 |
| Gallbladder Polys | Gallbladder Multiple Polyps (1) | 1 |
| Hepatic Hemangioma | Cavernous Hemangioma (4), Hemangioma with Arteriovenous Fistula (1) | 5 |
| Hepatic Cystic Lesions | Simple Hepatic Cyst (1), Multiple Hepatic Cystic Lesions (1), Hepatic Cyst (1) | 3 |
| Focal Nodular Hyperplasia (FNH) | Focal Nodular Hyperplasia (3) | 3 |
| Cirrhosis & Hepatic Steatosis | Nodular Cirrhosis (2), Fatty Liver (1) | 3 |
| Hepatic Abscess | Hepatic Abscess (1) | 1 |
| TOTAL |  | 24 |

**Table S2. Clinical characteristics of the four study cohorts**

|  |  | **Healthy** | **Benign** | **CCA** | **HCC** |
| --- | --- | --- | --- | --- | --- |
| n |  | 30 | 24 | 15 | 44 |
| Age(years), mean ± SD |  | 41.9 ± 10.7 | 51.7 ± 16.1 | 56.1 ± 8.9 | 58.1 ± 10.1 |
| Gender (M/F) |  | 18 / 12 | 13 / 11 | 8 / 7 | 41 / 3 |
| AFP | Negative (0-7 μg / L) | 30 | 22 | 12 | 19 |
|  | Positive (＞7 μg / L) | 0 | 2 | 3 | 25 |
| CEA | Negative (0-5 μg / L) | 30 | 24 | 8 | 40 |
|  | Positive (＞5 μg / L) | 0 | 0 | 7 | 4 |
| CA199 | Negative (0-35 μg / L) | 30 | 24 | 5 | 39 |
|  | Positive (＞35 μg / L) | 0 | 0 | 10 | 4 |
| ALT (U/L, mean ± SD) |  | 19.5 ± 9.3 | 91.7 ± 307.8 | 71.7 ± 121.5 | 42.9 ± 34.5 |
| AST (U/L, mean ± SD) |  | 21.8 ± 6.0 | 59.0 ± 169.8 | 53.6 ± 55.7 | 43.1 ± 32.1 |

**Abbreviations:**

M: Male;

F: Female;

SD: Standard deviation;

AFP: Alpha-fetoprotein;

CEA：Carcinoembryonic antigen;

CA199: Carbohydrate antigen199;

ALT: Alanine aminotransferase;

AST: Aspartate aminotransferase.

**Table S3. Sequence information was used in this study**

| **Name** | **Sequence (from 5’ to 3’)** |
| --- | --- |
| **Template strand** | AAGTATT/ACCAGAAAAAAGCUGUGTGTTGTAAGGGCCCGTGA  CTATGTCGAAGCGACCCGGCGATATAATCATTTCCACGCCCGTC |
| **Forward Primer** | TGTTGTAAGGGCCCGTGACT |
| **Reverse Primer** | GACGGGCGTGGAAATGATTA |
| **Probe** | CY5-TGTCGAAGCGACCCGGCGA-MGB |

**Table S4. 65 Parameters for machine learning**

| **Abbreviation** | **Full Name** | **Test Code** |
| --- | --- | --- |
| EV_13(S)-HODE | EV-derived 13(S)-Hydroxyoctadecadienoic acid | NA |
| Plasma_13(S)-HODE | Plasma-derived 13(S)-Hydroxyoctadecadienoic acid | NA |
| age | Age | NA |
| AFP | Alpha-fetoprotein | C0142 |
| CEA | Carcinoembryonic antigen | C0143 |
| CA199 | Carbohydrate antigen 19-9 | C0244 |
| ALT | Alanine aminotransferase | B0032 |
| AST | Aspartate aminotransferase | B0033 |
| AST/ALT Ratio | Aspartate aminotransferase to alanine aminotransferase ratio | NA |
| WBC | White blood cell count | A0125 |
| LYMPH# | Lymphocyte absolute count | A0126 |
| NEUT# | Neutrophil absolute count | A0128 |
| MONO# | Monocyte absolute count | A0130 |
| EOS# | Eosinophil absolute count | A0131 |
| BASO# | Basophil absolute count | A0132 |
| LYMPH% | Lymphocyte percentage | A0133 |
| NEUT% | Neutrophil percentage | A0135 |
| MONO% | Monocyte percentage | A0137 |
| EOS% | Eosinophil percentage | A0138 |
| BASO% | Basophil percentage | A0139 |
| RBC | Red blood cell count | A0140 |
| Hb | Hemoglobin | A0141 |
| HCT | Hematocrit | A0142 |
| MCV | Mean corpuscular volume | A0143 |
| MCH | Mean corpuscular hemoglobin | A0144 |
| MCHC | Mean corpuscular hemoglobin concentration | A0145 |
| RDW-SD | Red blood cell distribution width – standard deviation | A0147 |
| RDW-CV | Red blood cell distribution width – coefficient of variation | A0148 |
| PLT | Platelet count | A0149 |
| PCT | Platelet crit | A0150 |
| MPV | Mean platelet volume | A0151 |
| PDW | Platelet distribution width | A0153 |
| P-LCR | Platelet large cell ratio | A0154 |
| IG# | Immature granulocyte absolute count | A0383 |
| IG% | Immature granulocyte percentage | A0384 |
| HFLC% | High fluorescence lymphocyte cell percentage | A0385 |
| NEWX | New white blood cell parameter X | A0386 |
| NESFL | Neutrophil side fluorescence light | A0387 |
| LYZ | Lymphocyte side scatter | A0388 |
| MicroR | Microcyte ratio | A0389 |
| TCO2 | Total carbon dioxide | B0024 |
| UA | Uric acid | B0027 |
| Cr | Creatinine | B0029 |
| TP | Total protein | B0035 |
| ALB | Albumin | B0037 |
| Urea | Urea | B0039 |
| GLOB | Globulin | B0041 |
| A/G Ratio | Albumin to globulin ratio | B0042 |
| T-Bil | Total bilirubin | B0043 |
| D-Bil | Direct bilirubin | B0045 |
| I-Bil | Indirect bilirubin | B0047 |
| GLU | Glucose | B0063 |
| TG (1st) | Triglyceride | B0083 |
| TC (1st) | Total cholesterol | B0084 |
| HDL-C (1st) | High-density lipoprotein cholesterol | B0085 |
| LDL-C (1st) | Low-density lipoprotein cholesterol | B0086 |
| VLDL-C (1st) | Very low-density lipoprotein cholesterol | B0087 |
| eGFR (CKD-EPI) | Estimated glomerular filtration rate (CKD-EPI equation) | B0202 |
| Non-HDL-C (1st) | Non-high-density lipoprotein cholesterol | B0243 |
| TG (2nd) | Triglyceride | B0248 |
| TC (2nd) | Total cholesterol | B0249 |
| HDL-C (2nd) | High-density lipoprotein cholesterol | B0250 |
| LDL-C (2nd) | Low-density lipoprotein cholesterol | B0251 |
| VLDL-C (2nd) | Very low-density lipoprotein cholesterol | B0252 |
| Non-HDL-C (2nd) | Non-high-density lipoprotein cholesterol | B0253 |

**Table S5.  Top 15 most important parameters in the LightGBM model**

| **Importance** | **Split 1** | **Split 2** | **Split 3** |
| --- | --- | --- | --- |
| 1 | CA199 | CEA | CA199 |
| 2 | AFP | HDL-C | TP |
| 3 | PCT | ALB | AFP |
| 4 | CEA | AFP | LYMPH# |
| 5 | HDL-C | Plasma_13(S)-HODE | TG |
| 6 | Plasma_13(S)-HODE | CA199 | EV_13(S)-HODE |
| 7 | MONO# | D-Bil | NEWX |
| 8 | EV_13(S)-HODE | TC | Plasma_13(S)-HODE |
| 9 | MONO% | PCT | WBC |
| 10 | TG | TP | MONO# |
| 11 | ALB | MONO# | HDL-C |
| 12 | MCHC | HCT | RDW-CV |
| 13 | Hb | EV_13(S)-HODE | ALB |
| 14 | MicroR | NESFL | VLDL-C |
| 15 | LYMPH# | MCHC | MCHC |

**Abbreviations:**

CA199: Carbohydrate antigen199;

AFP: Alpha-fetoprotein;

PCT:Platelet crit;

CEA:Carcinoembryonic antigen;

HDL-C:High-density lipoprotein cholesterol

MONO#:Monocyte absolute count;

MONO%:Monocyte percentage;

TG:Triglyceride;

ALB:Albumin;

MCHC:Mean corpuscular hemoglobin concentration;

Hb:Hemoglobin;

MicroR:Microcyte ratio

LYMPH#:Lymphocyte absolute count;

D-Bil:Direct bilirubin;

TC:Total cholesterol;

TP:Total protein;

HCT:Hematocrit;

NESFL:Neutrophil side fluorescence light

NEWX:New white blood cell parameter X

WBC:White blood cell count;

RDW-CV:Red blood cell distribution width-coefficient of variation;

VLDL-C:Very low-density lipoprotein cholesterol.

**Table S6: Diagnostics performance of biomarker panel combine with 13S-HODE-EVs level with LightGBM model.**
